# Inhibition of SNW1 association with spliceosomal proteins promotes apoptosis in breast cancer cells

**DOI:** 10.1002/cam4.366

**Published:** 2014-12-01

**Authors:** Naoki Sato, Masao Maeda, Mai Sugiyama, Satoko Ito, Toshinori Hyodo, Akio Masuda, Nobuyuki Tsunoda, Toshio Kokuryo, Michinari Hamaguchi, Masato Nagino, Takeshi Senga

**Affiliations:** 1 Department of Surgical Oncology, Nagoya University Graduate School of Medicine65 Tsurumai, Showa, Nagoya, 466-8550, Japan; 2 Division of Cancer Biology, Nagoya University Graduate School of Medicine65 Tsurumai, Showa, Nagoya, 466-8550, Japan; 3 Division of Neurogenetics, Nagoya University Graduate School of Medicine65 Tsurumai, Showa, Nagoya, 466-8550, Japan

**Keywords:** Apoptosis, EFTUD2, PRPF8, RNA splicing, SNRNP200, SNW1

## Abstract

RNA splicing is a fundamental process for protein synthesis. Recent studies have reported that drugs that inhibit splicing have cytotoxic effects on various tumor cell lines. In this report, we demonstrate that depletion of SNW1, a component of the spliceosome, induces apoptosis in breast cancer cells. Proteomics and biochemical analyses revealed that SNW1 directly associates with other spliceosome components, including EFTUD2 (Snu114) and SNRNP200 (Brr2). The SKIP region of SNW1 interacted with the N-terminus of EFTUD2 as well as two independent regions in the C-terminus of SNRNP200. Similar to SNW1 depletion, knockdown of EFTUD2 increased the numbers of apoptotic cells. Furthermore, we demonstrate that exogenous expression of either the SKIP region of SNW1 or the N-terminus region of EFTUD2 significantly promoted cellular apoptosis. Our results suggest that the inhibition of SNW1 or its associating proteins may be a novel therapeutic strategy for cancer treatment.

## Introduction

Almost all the primary eukaryote transcripts are expressed as precursor mRNAs (pre-mRNAs) and converted to mRNAs by splicing, during which noncoding introns are removed and exons are joined. Splicing of pre-mRNAs is mediated by large protein-RNA complexes that consist of U1, U2, U5, and U4/U6 small nuclear ribonucleoproteins (snRNPs) and numerous additional proteins. Initially, the U1 and U2 snRNPs are assembled on the pre-mRNA, and U4/U6.U5 tri-snRNP, in which the U4 and U6 snRNAs are tightly base-paired, is recruited for spliceosome assembly. Subsequently, U1 and U4 snRNPs dissociated from the pre-mRNA, and the spliceosome becomes catalytically active for the intron removal and exon ligation. Once the exon ligation is complete, the spliceosome is disassembled, and snRNPs are recycled for the next round of splicing 1,2

PRPF8 (PRP8), SNRNP200 (Brr2), and EFTUD2 (Snu114) form a stable protein complex and are constitutive components of the U5 snRNP 3. PRPF8 is a highly conserved 280-kDa protein with no obvious homology to other proteins, and EFTUD2 is a GTPase protein homologous to eukaryotic translation initiation factor 2 (EF-2) 4,5. SNRNP200 is a RNA helicase that unwinds the U4/U6 snRNA duplex for the dissociation of U4 snRNP from the pre-mRNA 6. The complex is critical for the activation of the spliceosome, and mutations in these proteins are associated with genetic diseases, such as mandibulofacial dysostosis with microcephaly and retinitis pigmentosa 7,8. The helicase activity of SNRNP200 is regulated by both PRPF8 and EFTUD2. SNRNP200 activity is stimulated by GTP-loaded EFTUD2, but when bound to GDP, EFTUD2 represses SNRNP200 activity 9,10. The RNase H domain of PRPF8 inhibits loading of SNRNP200 to U4 snRNA, and a C-terminal part of PRPF8 modulates the SNRNP200 activity for the unwinding of U4/U6 snRNA duplex 11–13. Recent proteomics analyses have demonstrated that multiple proteins are in complex with these proteins; therefore, there may be additional proteins that directly associate with the complex for the regulation of the unwinding activity of SNRNP200 14,15.

SNW1/SKIP is a highly conserved protein associated with splicing and transcription. Prp45p, a yeast homolog of SNW1, interacts with other splicing factors, such as Prp22p and Prp46p, and its depletion causes defects in splicing 16. Human SNW1 is recruited to the pre-mRNA when the U1 and U4 snRNPs dissociate from the spliceosome 17. SNW1 promotes the recruitment of U2AF65 to the pre-mRNA for the splicing and expression of p21, a target protein of p53 18. The critical function of SNW1 in transcription is supported by the ability of SNW1 to associate with numerous transcriptional factors to modulate their activities. SNW1 has been described as a coactivator of Notch and nuclear receptors, such as the vitamin D receptor, retinoic acid receptor, and androgen receptor 19–23. In addition, SNW1 associates with P-TEFb, c-myc, and Menin to activate the HIV-1 promoter 24,25.

A previous study using a genome-scale library of endoribonuclease-prepared siRNAs revealed that depletion of SNW1 promoted defects in cell division 26. Proliferating cancer cells are generally sensitive to drugs that inhibit cell division; thus, these drugs are used for cancer treatment. We speculate that the inhibition of SNW1 function would promote cancer cell apoptosis and that SNW1 could be a novel therapeutic target for cancer treatment. In this report, we demonstrate that SNW1 directly associates with EFTUD2 and SNRNP200 and that disruption of SNW1 association with these proteins promotes the apoptosis of breast cancer cells.

## Materials and Methods

### Cells and antibodies

All cell lines were maintained in Dulbecco's Modified Eagle's medium (DMEM) supplemented with 10% Fetal bovine serum (FBS). The anti-Flag antibody was obtained from Wako (Osaka, Japan), the anti-GFP antibody from NeuroMab (Davis, CA), the anti-SNW1 antibody from Sigma-Aldrich (St. Louis, MO), the anti-PRPF8 and anti-EFTUD2 antibodies from GeneTex (Hsinchu, Taiwan), and the anti-SNRNP200 antibody from Bethyl Laboratories (Montgomery, TX). The anti-cleaved PARP antibody was purchased from Cell Signaling (Danvers, MA).

### cDNA constructs

cDNAs for human SNW1, EFTUD2, SNRNP200, and PRPF8 were amplified by PCR from a cDNA library of HeLa cells. Each cDNA was cloned into the pQCXIP vector (TAKATA, Tokyo, Japan) with an N-terminal Flag or GFP tag. Deletion mutants were generated by PCR.

### Mass spectrometry analysis

293T cells that constitutively express Flag-SNW1 were generated by retrovirus infection. The pQCXIP vector encoding Flag-SNW1 was transfected into 293T cells in combination with the pVPack-GP and pVPack-Ampho vectors (Stratagene, Tokyo, Japan) using Lipofectamine 2000 (Invitrogen, Carlsbad, CA). Forty-eight hours after transfection, the supernatants were added to 293T cells with 2 *μ*g/mL polybrene (Sigma-Aldrich), and infected cells were selected with 1 *μ*g/mL puromycin for 3 days. The Flag-SNW1-expressing 293T cells were lysed and immunoprecipitated with an anti-Flag antibody and immunoprecipitates were mixed with a Flag peptide to elute immunoprecipitated proteins. The eluted proteins were digested with trypsin and subjected to mass spectrometry analysis using the LC-MS/MS system (Paradigm MS4, Michrom Bioresources, Sacramento, CA; HTS-PAL, CTC Analytics AG, Zwingen, Swiss; LTQ Orbitrap XL, Thermo Scientific, Waltham, MA USA). The proteins were identified using the Mascot software package (Matrix Science, London, UK).

### siRNA transfection

The sequences of the siRNAs used to suppress SNW1 expression were 5′-GGAGGUUAUGAAUGCAGAUTT-3′ (SNW1-1) and 5′-CCCUAAUGAUGCAAGUCAUTT-3′ (SNW1-2). The sequence of the siRNA for EFTUD2 depletion was 5′-GGAAGAAGCUGGGAGAGUUTT-3′. The sequence of the control siRNA targeting luciferase was 5′-CUUACGCUGAGUACUUCGATT-3′. siRNAs were obtained from Sigma-Aldrich. Cells were transfected with 20 nmol/L of siRNA using Lipofectamine RNAiMAX (Invitrogen) according to the manufacturer's instructions.

### Proliferation assay

siRNA-transfected cells were cultured in 96-well plates, and the number of viable cells at the indicated time points were evaluated using the Cell Count Kit 8 (Dojindo, Tokyo, Japan).

### TUNEL assay

Cells were transfected with siRNAs and 72 h later, the cells were subjected to TUNEL assay using the In Situ Cell Death Detection Kit (Roche, Basel, Switzerland) according to the manufacturer's protocol. Cells in five randomly selected fields were evaluated, and three independent experiments were performed.

### Transfection and immunoprecipitation

To detect association of Flag-SNW1 with GFP-EFTUD2, GFP-SNRNP200, and GFP-PRPF8, 293T cells cultured in 12-well plates were transfected with 0.5 *μ*g of each plasmid using Lipofectamine 2000. After 24 h, cells were lysed with lysis buffer with protease inhibitors and immunoprecipitated with an anti-Flag antibody. The precipitates were washed with lysis buffer four times and suspended with Laemmli sample buffer for immunoblot analysis. Other experiments for interactions were performed in the same procedure.

### In vitro translation and pull down assay

cDNAs were cloned into the pcDNA3.1 vector (Invitrogen) with an N-terminal HA tag, and in vitro translation was performed using TNT Quick Coupled Transcription/Translation System (Promega, Madison WI) according to the manufacturer's protocol. GST-fused SNW1-174-335 bound to glutathione agarose beads were mixed with in vitro-translated proteins for 1 h and affinity precipitated. The precipitates were washed with lysis buffer four times and suspended with Laemmli sample buffer for immunoblot analysis.

### Time-lapse analysis

Cells cultured on glass-based dishes were transfected with siRNAs or plasmids. After 24 h, the cells were monitored for 48 h using a time-lapse microscope system (LCV110; Olympus, Tokyo, Japan). Images were acquired and analyzed using the MetaMorph Imaging System (Universal Imaging, Silicon Valley, CA).

## Result

### SNW1 depletion promote apoptosis

**Figure 1:**
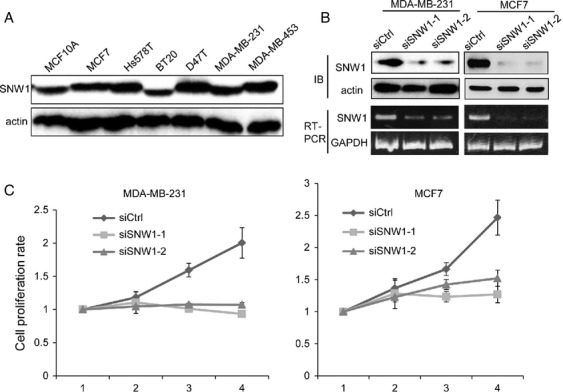
Depletion of SNW1 suppresses cell proliferation. (A) The expression of SNW1 in breast cancer cell lines was determined by immunoblot. (B) MDA-MB-231 and MCF7 cells were transfected with siRNAs. After 72 h, level of SNW1 protein or SNW1 mRNA was determined by immunoblot or RT-PCR. (C) Cells were transfected with siRNAs, and the number of viable cells at the indicated time points was evaluated by the Cell Counting Kit 8.

**Figure 2:**
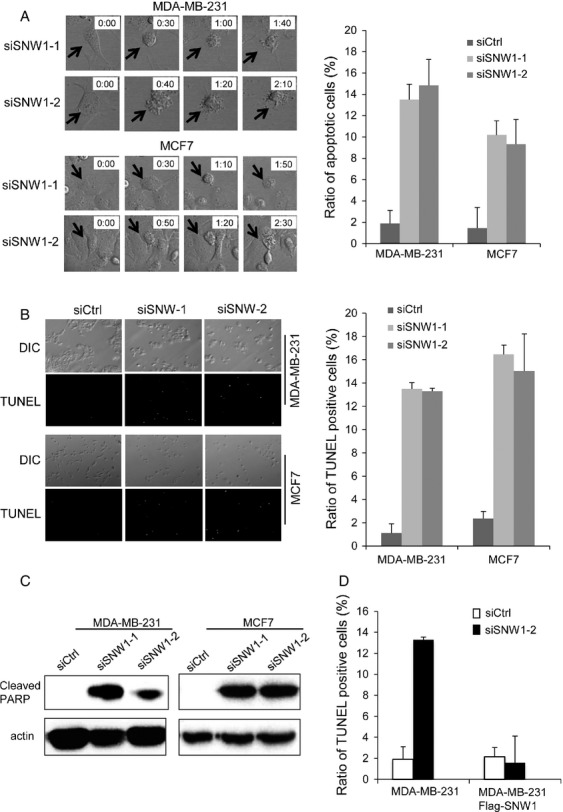
Depletion of SNW1 promotes apoptosis. (A) Cells were transfected with siRNAs, and after 24 h, the cells were observed using time-lapse microscopy for 48 h. Representative pictures are presented; the arrows indicate apoptotic cells. The graph presents the ratio of cells undergoing apoptosis. At least 150 cells were observed from three independent experiments. (B) Cells were transfected with siRNAs, and after 72 h, the cells were subjected to TUNEL assay. Representative pictures are presented. The graph presents the ratio of TUNEL-positive cells. (C) Cells were transfected with siRNAs, and after 72 h, cells were lysed and immunoblotted for cleaved PARP. (D) MDA-MB-231 cells that constitutively expressed SNW1 were established by retrovirus infection. Parental and SNW-expressing MDA-MB-231 cells were transfected with siRNAs. After 72 h, cells were subjected to TUNEL assay. The graph presents the ratio of TUNEL-positive cells.

To determine whether SNW1 is essential for the proliferation and survival of cancer cells, we used breast cancer cells. SNW1 was expressed in all of the breast cancer cells we examined and normal mammary epithelial cells, MCF10A (Fig.1A). We depleted SNW1 in MDA-MB-231 and MCF7 cells and examined the effects on cell proliferation. Two siRNAs targeting different regions of SNW1 efficiently depleted SNW1 expression in both cell lines (Fig.1B). Cells were transfected with siRNAs and subjected to cell proliferation assays. Depletion of SNW1 by either siRNA significantly inhibited the proliferation of MDA-MB-231 and MCF7 cells (Fig.1C). To explore how the cell proliferation was inhibited, we used time-lapse microscopy to observe SNW1-depleted cells. A number of SNW1-depleted cells formed membrane blebs and shrank and eventually died (Fig.2A). Approximately 10–15% of SNW1-knockdown cells died, whereas most of the control siRNA-transfected cells exhibited no changes in cellular morphology (Fig.2A). As the changes in morphology were indicative of apoptosis, we performed a TUNEL assay, which detects fragmented DNA induced during the course of apoptosis. MDA-MB-231 and MCF7 cells were transfected with siRNAs and 48 h later, cells were subjected to the TUNEL assay. As shown in Figure2B, a significantly increased number of SNW1-depleted cells were apoptotic compared with control siRNA-transfected cells. To further confirm induction of apoptosis by SNW1 knockdown, we examined the expression of cleaved PARP. Immunoblot analysis with an anticleaved PARP antibody revealed elevated levels of cleaved PARP in SNW1-knockdown cells (Fig.2C). We performed rescue experiments to confirm that the induced apoptosis was specifically dependent on the suppression of SNW1 expression. MDA-MB-231 cells that constitutively expressed Flag-tagged SNW1 were established by retrovirus infection, and the cells were transfected with control siRNA or SNW1 siRNA2 that targeted the 3′UTR of SNW1 mRNA. TUNEL assay revealed that the Flag-SNW1-expressing cells were resistant to the induction of apoptosis by SNW1 siRNA2-transfection (Fig.2D). These results indicate that the depletion of SNW1 in breast cancer cells promotes apoptosis.

### SNW1 directly associates with EFTUD2 and SNRNP200

**Figure 3:**
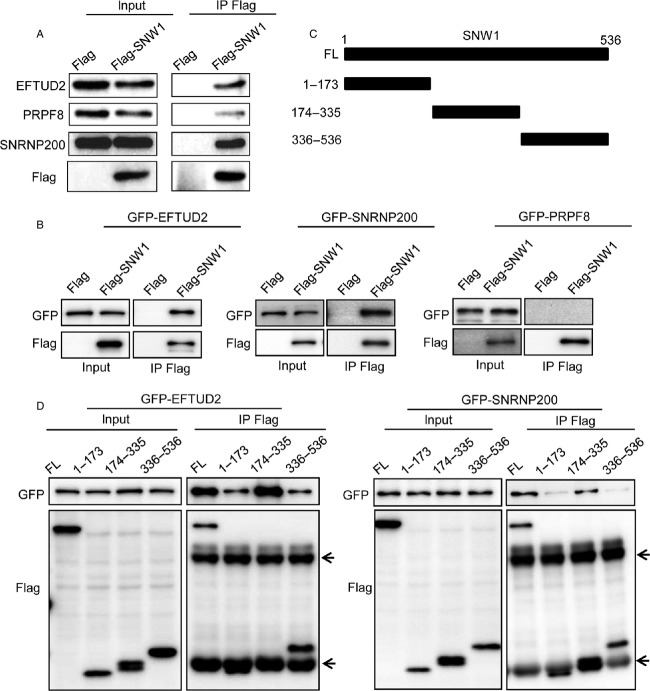
The SKIP domain of SNW1 associates with EFTUD2 and SNRNP200. (A) Flag-SNW1- or Flag-expressing 293T cells were lysed and immunoprecipitated with an anti-Flag antibody. The immunoprecipitates were subjected to immunoblot analysis with the indicated antibodies. (B) Flag-SNW1 was expressed in 293T cells together with either GFP-EFTUD2, GFP-SNRNP200 or GFP-PRPF8, and after 24 h, cells were lysed and immunoprecipitated with anti-Flag antibody. The immunoprecipitates were immunoblotted with an anti-GFP or anti-Flag antibody. (C) Schematic representation of the deletion constructs of SNW1. (D) GFP-EFTUD2 or GFP-SNRNP200 was expressed in 293T cells together with deletion mutants of Flag-tagged SNW1. After 24 h, the cells were lysed and immunoprecipitated with an anti-Flag antibody. The immunoprecipitates were immunoblotted with an anti-GFP or anti-Flag antibody. The arrows indicate the heavy chain and light chain of the antibody.

To obtain a clue as to how SNW1-depletion-induced apoptosis, we performed mass spectrometry analysis to identify SNW1-interacting proteins. 293T cells that constitutively express Flag-SNW1 were established by retrovirus infection, and Flag-SNW1 and its associating proteins were immunoprecipitated. The immunoprecipitated protein complex was eluted and subjected to mass spectrometry analysis. Consistent with the previous proteomics analysis 15, we obtained various proteins involved in RNA splicing. Among these proteins, PRPF8, SNRNP200, and EFTUD2 were identified with relatively high scores (Table S1). Three of these proteins are known to associate with each other and are essential for the process of RNA splicing. We first tested whether Flag-SNW1 was in complex with these proteins by immunoblot. 293T cells constitutively expressing either Flag tag or Flag-SNW1 were immunoprecipitated with an anti-Flag antibody and then immunoblotted with antibodies for PRPF8, SNRNP200, and EFTUD2. As Figure3A demonstrates, all of these endogenous proteins were specifically precipitated with Flag-SNW1. To determine whether each of these proteins directly associates with SNW1, we expressed Flag-SNW1 together with GFP-tagged PRPF8, SNRNP200, or EFTUD2 in 293T cells and examined their interactions. Although exogenously expressed GFP-SNRNP200 and GFP-EFTUD2 were clearly coprecipitated with Flag-SNW1, GFP-PRPF8 was not precipitated with Flag-SNW1 (Fig.3B). These results suggest a direct interaction of EFTUD2 and SNRNP200 with SNW1 in cells. To determine which region of SNW1 was critical for the interaction, the deletion constructs depicted in Figure3C were created. SNW1 has a SKIP domain (aa174-335) in the central region, and there are no other specific domains. We examined the association of these deletion constructs of SNW1 and EFTUD2 as well as SNRNP200. Each Flag-tagged SNW1 mutant was expressed in 293T cells together with GFP-tagged EFTUD2 or SNRNP200 and was immunoprecipitated with an anti-Flag antibody followed by immunoblot with an anti-GFP antibody. The SKIP region was essential for the association, and neither the N-terminus nor C-terminus without the SKIP region was coprecipitated with EFTUD2 or SNRNP200 (Fig.3D).

**Figure 4:**
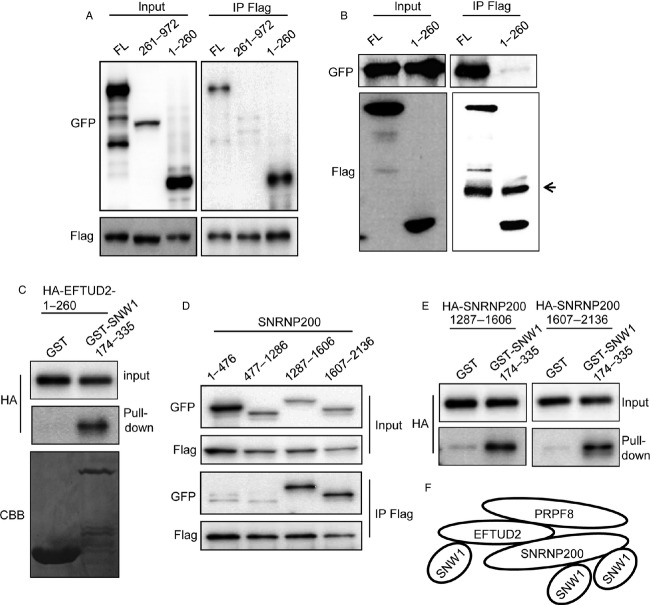
The N-terminus of SNW1 and the C-terminus of SNRNP200 directly associate with the SKIP domain of SNW1. (A) The interaction of Flag-SNW1 with full-length as well as deletion mutants of GFP-tagged EFTUD2 in 293T cells was examined by immunoprecipitation with an anti-Flag antibody. (B) The interaction of GFP-SNRNP200 with Flag-tagged full-length or 1-260 EFTUD2 in 293T cells was examined by immunoprecipitation with an anti-Flag antibody. The arrow indicates the heavy chain of the antibody. (C) In vitro-translated HA-tagged EFTUD2-1-260 was mixed with GST or GST-fused SNW1-174-335 bound to glutathione agarose beads and affinity precipitated. The precipitates were immunoblotted with the indicated antibodies. (D) The interaction of Flag-SNW1 with GFP-tagged deletion mutants of SNRNP200 in 293T cells was examined by immunoprecipitation with an anti-Flag antibody. (E) In vitro-translated HA-tagged deletion mutants of SNRNP200 were mixed with GST or GST-fused SNW1-174-335 bound to glutathione agarose beads and affinity precipitated. The precipitates were immunoblotted with the indicated antibodies. (F) Schematic representation of SNW1 association with EFTUD2 and SNRNP200.

We next investigated which regions of EFTUD2 were responsible for the association with SNW1. EFTUD2 was divided into two regions, 1-260 and 261-972, and their interactions with SNW1 in cells were examined by immunoprecipitation. As shown in Figure4A, the 1-260 region of EFTUD2 associated with SNW1. To exclude the possibility that the association of SNW1 and EFTUD2-1-260 is mediated by SNRNP200, we examined the association of EFTUD2-1-260 and SNRNP200. Although SNRNP200 clearly associated with full-length EFTUD2, SNRNP200 was not coprecipitated with EFTUD2-1-260 (Fig.4B). To confirm the direct interaction, we used a recombinant protein. GST-fused SNW1-174-335 (SKIP domain) bound to glutathione beads was mixed with in vitro-translated EFTUD2-1-260, and associating proteins were precipitated. As shown in Figure4C, EFTUD2-1-260 directly interacted with the SKIP domain of SNW1. We next examined whether the SKIP domain also directly associated with SNRNP200. Because SNRNP200 is a large protein whose molecular weight is greater than 200 kDa, we divided the protein into four regions. Deletion constructs of GFP-tagged SNRNP200 were expressed in 293T cells together with Flag-SNW1 and immunoprecipitated with an anti-Flag antibody. Immunoprecipitation analysis revealed that two regions in the C-terminus of SNRNP200, 1287-1606, and 1607-2136, associated with SNW1 (Fig.4D). Direct interaction of each region with the SKIP domain was confirmed using in vitro-translated proteins and GST-SKIP (Fig.4E). These results demonstrate that SKIP domain associates with multiple sites, including N-terminus of EFTUD2 and two regions of SNRNP200 in the C-terminus (Fig.4F).

### Exogenous expression of deletion proteins for SNW1 and EFTUD2 promote apoptosis

**Figure 5:**
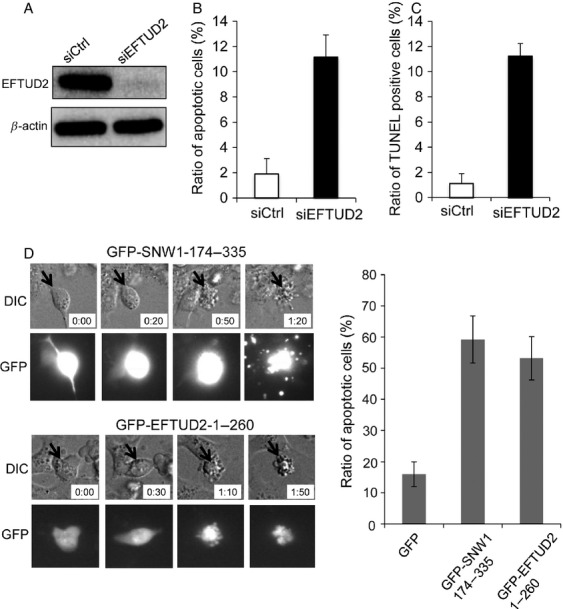
The expression of deletion mutants of SNW1 or EFTUD2 promotes apoptosis. (A) MDA-MB-231 cells were transfected with siRNAs, and after 72 h, the expression of EFTUD2 was determined by immunoblot. (B) MDA-MB-231 cells transfected with siRNAs were observed using time-lapse microscopy, and apoptotic cells were evaluated. The graph presents the ratio of cells undergoing apoptosis. (C) MDA-MB-231 cells transfected with siRNAs were subjected to TUNEL assay. The graph presents the ratio of TUNEL-positive cells. (D) MDA-MB-231 cells were transfected with plasmid encoding either GFP-SNW1-174-335 or GFP-EFTUD2-1-260, and after 24 h, cells were observed using time-lapse microscopy for 48 h. Representative images are presented. The graph indicates the ratio of GFP-positive cells that became apoptotic. At least 80 cells were observed in each transfection from three independent experiments.

We tested whether EFTUD2, a direct interacting protein of SNW1, was required for the survival of MDA-MB-231 cells. Transfection of siRNA sufficiently depleted EFTUD2 expression in cells (Fig.5A). Similar to the depletion of SNW1, time-lapse analysis revealed that more than 10% of EFTUD2-knockdown cells underwent morphological changes indicative of apoptosis (Fig.5B). TUNEL assay confirmed the increase in apoptotic cells by EFTUD2 depletion (Fig.5C). Finally, we expressed deletion mutants of SNW1 and EFTUD2 to disrupt the complex formation of endogenous proteins and examined its effect. MDA-MB-231 cells were transfected with plasmids encoding GFP, GFP-SNW1-174-335, or GFP-EFTUD2-1-260. After 24 h, cells were observed by time-lapse microscopy. Transfection of GFP alone induced some apoptotic cells, possibly due to the toxic effect of the transfection reagent. Surprisingly, more than 50% of cells that expressed either GFP-SNW1-174-335 or GFP-EFTUD2-1-260 became apoptotic (Fig.5D). These results suggest that disruption of SNW1 association with the spliceosome promotes apoptosis.

## Discussion

In this report, we demonstrate that depletion of SNW1 and its associating factor, EFTUD2, induced apoptosis in breast cancer cells. In addition, the expression of a deletion construct of either SNW1 or EFTUD2, which can inhibit the association of endogenous proteins, significantly increased the number of apoptotic cells. These results indicate that SNW1 recruitment to the U5 snRNP is essential for the splicing process and cell survival. Previous studies have demonstrated that the depletion of various splicing factors induces defects in mitosis 27. Consistent with the previous study, we observed an increase in mitotic cells when HeLa cells were transfected with siRNAs for SNW1 and EFTUD2 (Fig. S1); however, we did not observe mitotic arrest in either MDA-MB-231 cells or MCF7 cells. It appears that the phenotype induced by disruption of RNA splicing may be dependent on cell type.

Recent studies have revealed that drugs that inhibit spliceosome function have cytotoxic effects on cancer cells. Spliceostatin A, GEX1A, and pladienolide B are natural substances that exhibit cytotoxic effects on multiple tumor cell lines 28. Affinity precipitation analysis revealed that splicing factor 3B (SF3B), which is a subcomplex of U2 snRNP and is composed of five proteins, was the target component of the compounds 29,30. Spliceostatin A prevents the binding of SF3B to the pre-mRNA and subsequently inhibits spliceosome assembly or induces changes in alternative splicing 31. The defect in splicing process by spliceostatin A affected the expression of key regulators for cell division, such as Cyclin A and Aurora A kinase, thereby explaining the mechanisms of antitumor effect of the compound 32. These studies have demonstrated that inhibition of the splicing process can be a novel target for cancer treatment. Considering the critical function of SNW1 and its associating proteins for cell survival, drugs that can inhibit the function of the SNW1 complex may be useful for cancer treatment. However, it has to be reminded that induction of apoptosis by SNW1 or EFTUD2 knockdown was not specific to cancer cells because depletion of either protein in normal mammary epithelial cells, MCF10A, also induced significant apoptosis (data not shown).

SNRNP200 is a critical RNA helicase that unwinds the U4/U6 snRNA duplex. Although it has been demonstrated that the PRPF8 RNase H domain inhibits SNRNP200 activity and GTP-bound EFTUD2 and that the C-terminus of PRPF8 induces the activation of SNRNP200 10–13, the detailed molecular mechanisms of the activation of SNRNP200 remains to be determined. We demonstrated that the SKIP domain of SNW1 can directly associate with the N-terminus of EFTUD2 and two independent region of SNRNP200. SNRNP200 has two Sec63 domains in the C-terminus. Both deletion proteins of SNRNP200 that interacted with SNW1 contained Sec63 domains, thus, the SKIP domain may directly associate with the Sec63 domain of SNRNP200. SNW1 is recruited to the U5 snRNP when the unwinding of U4/U6 snRNA duplex starts; thus, SNW1 association may be essential for the helicase activity of SNRNP200 17. In addition to the PRPF8/EFTUD2/SNRNP200 complex, our proteomics analysis as well as previous analyses found that SNW1 was in complex with another splicing subcomplex, PRP19 complex. The PRP19 complex is present in U5 snRNP and is essential for the activation and rearrangement of the spliceosome 33. However, the detailed molecular mechanisms of PRP19 function remain uncertain. Further studies on SNW1 and its associating proteins may provide novel insight into the molecular basis of the splicing process and therapeutic strategies in cancer treatment.

## Supporting Information

**Figure S1.** Depletion of SNW1 or EFTUD2 in HeLa cells induces mitotic arrest.

**Table S1.** Proteins coprecipitated with Flag-SNW1 in 293T cells were identified by mass spectrometry analysis.
