# Supplementary material for: Inhibition of SNW1 association with spliceosomal proteins promotes apoptosis in breast cancer cells
Source: Cancer Med. 2014 Dec 1;4(2):268–77. doi: 10.1002/cam4.366 (PMC4329010; doi:10.1002/cam4.366)
Supplement: Supplementary file 1 [file cam40004-0268-sd1.pdf]

Fig. S1

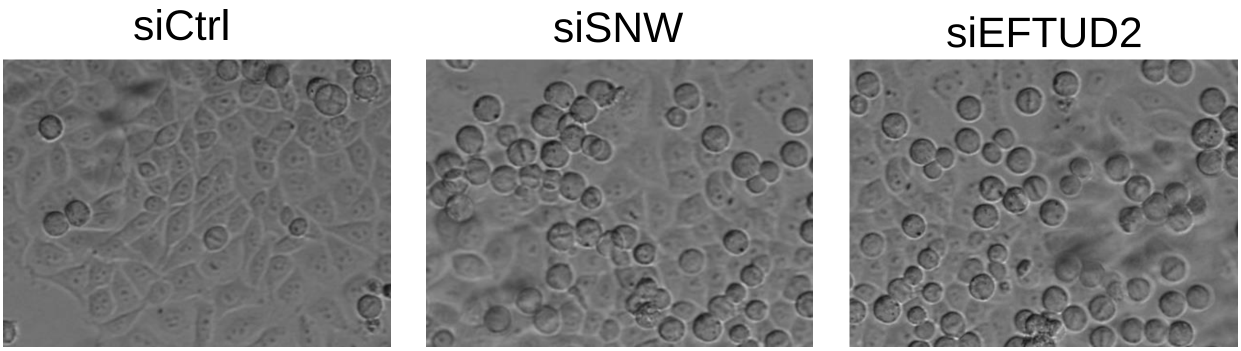

Figure S1. Depletion of SNW1 or EFTUD2 in HeLa cells induces mitotic arrest. HeLa cells were transfected with siRNAs and 72 h later, images were taken by microscope. Representative pictures are shown.
